# Genetic polymorphisms in genes associated with drug resistance in *Plasmodium vivax* parasites from northeastern Myanmar

**DOI:** 10.1186/s12936-022-04084-y

**Published:** 2022-03-03

**Authors:** Fang Huang, Shigang Li, Peng Tian, Lahpai Ja Seng Pu, Yanwen Cui, Hui Liu, Lianzhi Yang, Dahidam Yaw Bi

**Affiliations:** 1 National Institute of Parasitic Diseases, Chinese Center for Disease Control and Prevention, Shanghai, China; 2 Chinese Center for Tropical Diseases Research, Shanghai, China; 3 NHC Key Laboratory of Parasite and Vector Biology, Shanghai, China; 4 WHO Collaborating Centre for Tropical Diseases; National Center for International Research on Tropical Diseases, Shanghai, China; 5 Yingjiang County Center for Disease Control and Prevention, Yingjiang, Yunnan China; 6 Yunnan Institute of Parasitic Diseases, Pu’er, Yunnan China; 7 Laiza Central Civilian Hospital, Laiza, Myanmar; 8 Nabang Township Hospital, Yingjiang, Yunnan China; 9 Laiza Central Ministry of Public Health, Laiza, Myanmar

**Keywords:** Genetic polymorphisms, Drug resistance, *Plasmodium vivax*, Northeastern Myanmar

## Abstract

**Background:**

Anti-malarial drug resistance is still a major threat to malaria elimination in the Great Mekong Sub-region. *Plasmodium vivax* parasites resistant to anti-malarial drugs are now found in Myanmar. Molecular surveillance on drug resistance genes in *P. vivax* parasites from northeastern Myanmar was aimed at estimating the underlying drug resistance in this region.

**Methods:**

Blood samples from patients with vivax malaria were collected from Laiza city in northeastern Myanmar in 2020. Drug resistance genes including *Pvcrt-o, Pvmdr1*, *Pvdhfr* and *Pvdhps* were amplified and sequenced. Genetic polymorphisms and haplotypes were analysed to evaluate the prevalence of mutant alleles associated with drug resistance.

**Results:**

A total of 149 blood samples from *P. vivax* patients were collected. The prevalence of *Pvmdr1* mutations at codons 958 and 1076 was 100.0% and 52.0%, respectively, whereas no single nucleotide polymorphism was present at codon 976. The proportions of single and double mutant types were 48.0% and 52.0%, respectively. A K10 “AAG” insertion in the *Pvcrt-o* gene was not detected. Mutations in *Pvdhfr* at codons 57, 58, 61, 99 and 117 were detected in 29.9%, 54.3%, 27.6%, 44.9% and 55.1% of the samples, respectively. Wild type was predominant (46.3%), followed by quadruple and double mutant haplotypes. Of three types of tandem repeat variations of *Pvdhfr*, Type B, with three copies of GGDN repeats, was the most common. *Pvdhps* mutations were only detected at codons 383 and 553 and the wild type *Pvdhps* was dominant (78.0%). Eleven haplotypes were identified when combining the mutations of *Pvdhfr* and *Pvdhps*, among which the predominant one was the wild type (33.9%), followed by double mutant alleles S58R/S117N /WT (24.6%).

**Conclusions:**

This study demonstrated resistant *P. vivax* phenotypes exists in northeastern Myanmar. Continued surveillance of drug resistance markers is needed to update treatment guidelines in this region.

**Supplementary Information:**

The online version contains supplementary material available at 10.1186/s12936-022-04084-y.

## Background

*Plasmodium vivax*, the most geographically widespread human malaria parasite, causes significant morbidity in Southeast Asia, the Western Pacific, Central and South America, and parts of Africa [1]. There were an estimated 2.5 billion people at risk of *P. vivax* infection worldwide and 6.4 million clinical vivax malaria cases in 2019, mainly distributed in Southeast Asia [2]. In the past 20 years, the burden of *P. vivax* malaria has decreased dramatically, with many countries in the Asia-Pacific and the Americas reporting reductions of 90% in the number of clinical cases [2]. As a consequence, 34 countries are actively attempting to eliminate malaria, and countries in Central America and East Asia have declared their intention to eliminate malaria from their regions by 2025 and 2030, respectively [3, 4].

Myanmar, formerly Burma, has the heaviest malaria burden in the Greater Mekong Sub-region (GMS), with more cases and deaths than the rest of the region combined [2]. In recent years, Myanmar has made significant progress in reducing malaria morbidity and mortality [5–7]. Driven by the emergence and spread of artemisinin resistance in the GMS, the World Health Organization (WHO) declared their goal to eliminate malaria in the GMS by 2030 [8]. The National Plan for Malaria Elimination in Myanmar 2016–2030 was developed with the goal of decreasing the annual parasite index (API) to < 1 in all states/regions by 2020, interrupting the transmission of falciparum malaria in all states/regions by 2025 and eliminating malaria nationwide by 2030 [5]. However, conflict-affected settings and regions with high population mobility have posed challenges to malaria elimination [9].

*Plasmodium vivax* resistance to different anti-malarial drugs, including chloroquine (CQ), mefloquine, sulfadoxine-pyrimethamine (SP), has been reported in many countries or regions [10–15]. Molecular surveillance of drug resistance markers, as one of the tools in malaria surveillance, has been widely performed throughout the world [16]. Several candidate drug resistance genes in *P. vivax* parasites have been identified [17]. *Plasmodium vivax* chloroquine resistance transporter (*Pvcrt*) and the *P. vivax* multidrug resistance transporter (*Pvmdr1*) have been confirmed to be the *Plasmodium falciparum* orthologs involving CQ resistance genes [18, 19]. The *Pvcrt-o* gene was characterized nearly two decades ago [19]; it is similar to its *P. falciparum* orthologue and has an intron-rich gene structure with 14 exons that encodes a protein with 451 amino acids and 10 membrane domains [20]. Sequence polymorphism is relatively limited in the *Pvcrt-o* locus but a lysine (AAG) insertion at amino acid position 10 (K10), which was initially discovered in Southeast Asian strains, has been suggested to be associated with CQ resistance [21]. Since then, this polymorphism has been observed in both Southeast Asian and South American parasites [17, 22, 23]. The *Pvmdr1* gene encodes a transmembrane protein of the parasite’s digestive vacuole with 12 transmembrane domains and 1464 amino acids, and was characterized in 2005 [24].

Sequencing of *Pvmdr1* genes from several regions of the world has revealed more than fifty polymorphisms, as well as copy number variants (CNVs). Six SNPs have been reported at high frequency in multiple studies in regions with reported drug resistance: S513R, G698S, M908L, T958M, Y976F and F1076L. Among these, the two most common mutations were Y976F and F1076L. The essential enzymes dihydropteroate synthase (DHPS) and dihydrofolate reductase-thymidylate synthase (DHFR-TS) are involved in folate synthesis and are targets of sulfadoxine and pyrimethamine, respectively [14, 25]. SP has been used to treat *P. falciparum* for a long time, but it is primarily used for intermittent preventive treatment in pregnancy (IPTp) and IPT for infants (IPTi), and seasonal malaria chemoprevention combining with amodiaquine in some regions of Africa owing to its widespread resistance [26–28].

CQ is also used to treat vivax malaria in Southeast Asia, the prevalence of high CQ-resistant falciparum malaria resulted for a time in the widespread use of SP, which was available widely in areas where malaria was endemic and was still a first-line treatment in the adjacent countries Laos and Myanmar [29]. However, CQ and SP resistant *P. falciparum* and *P. vivax* were reported in Myanmar several decades ago, especially in southern Myanmar and at the border between Myanmar and Thailand [29–35]. Recently, increasing clinical failures after CQ treatment have been reported in multiple regions of Myanmar [36, 37]. Currently, the first-line treatment for *P. vivax* in Myanmar is CQ combined with primaquine (PQ) [5]. In northeastern Myanmar, the treatment efficacy of CQ was found to be relatively high, with a cumulative rate of parasite recurrence less than 3% [35]. The northernmost state in Myanmar, with a long border of almost 2000 km with Tibet and Yunnan Province in China, mainly in Kachin State, also called Jinghpaw Mung, where malaria has a relatively high rate of transmission [6], resulting from relatively low access to health services and difficulties in deploying interventions to hard-to-reach communities [38, 39]. Limited data have defined the molecular epidemiology of *P. vivax* resistance markers in northeastern Myanmar. In this study, genetic polymorphisms in genes potentially associated with drug resistance in *P. vivax* parasites from northeastern Myanmar were analysed to estimate the underlying drug resistance, with the aim of implementing an appropriate drug policy in this region.

## Methods

### Study site

**Fig. 1:**
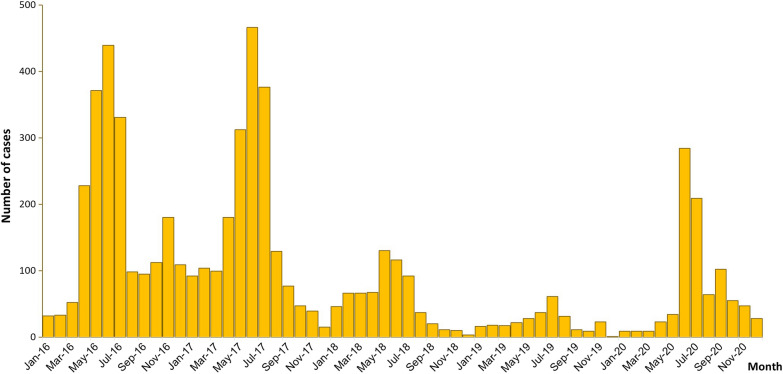
Monthly reported cases of malaria from sentinel sites in Laiza in Kachin State, Myanmar, 2016–2020

Samples were collected from central hospitals and community clinics in Laiza (24° 45′ 36″ N, 97° 33′ 48″ E), a remote city in Kachin State in northeastern Myanmar, along the China-Myanmar border. This region is mountainous and its climate is defined as subtropical, with a dry season from October to April and a rainy season from May to September. In recent years, malaria cases have declined dramatically owing to cooperation between China and Myanmar towards malaria elimination [38, 40]. Local malaria transmission displayed a distinct seasonality with two peaks, a large peak in April-August and a small peak in November, except 2020 with a mall peak in September (Fig. 1). Four human malaria parasites including *P. falciparum*, *P. vivax*, *Plasmodium ovale* and *Plasmodium malariae* have been detected, and *P. vivax* is the predominant species, accounting for more than 90% [41]. On the other side of Laiza city is Nabang township, Yunnan, China, and there is no barrier along the border. A large number of immigrants from both countries cross the border frequently through Nabang port, one of the provincial ports along the border. *Anopheles minimus* is reported to be the dominant mosquito species in this area and most malaria infection occurred in population with subsistence activities associated with forest areas, such as logging, banana or rubber planting, and living in planting areas during the farming season or entire year [42].

### Sample collection and DNA extraction

Blood samples were collected from *P. vivax*-infected patients prior to anti-malarial drug treatment in the central hospitals and community clinics in Laiza city from July to October 2020. Blood samples were spotted on filter paper (Whatman™ 903, GE Healthcare, USA), air dried, and then stored at − 20 °C before DNA extraction. Parasite DNA was extracted from the dried blood spots using a QIAamp DNA Mini Kit (Valencia, CA, USA) following the protocol. A polymerase chain reaction (PCR) amplifying the small-subunit rRNA gene of *Plasmodium* spp. [43] was performed to confirm the positive samples and identify the species before the four genes were sequenced.

### PCR amplification and sequencing

The *Pvcrt-o* gene was amplified by a single-round PCR and *Pvmdr1*, *Pvdhps* and *Pvdhfr* were amplified by nested PCR, as previously described [21, 34, 44–46]. The primers, cycling conditions and sizes of the PCR products are shown in Additional file 1. PCR reactions were performed in a 25 µL reaction mixture that contained 1 µL of genomic DNA, 12.5 µL 2×Taq Master Mix (TIANGEN, Beijing, China), 9.5 µL ddH_2_O and 10 µM primers. The amplification products were analysed by 1.5% agarose gel electrophoresis and directly sequenced. The PCR products were purified using filter plates (Edge Biosystems, Gaithersburg, MD, USA) and sequenced on an ABI 3730XL automatic sequencer. Bidirectional sequencing was performed, and all the products were sequenced twice using independently amplified PCR products.

### Data analysis

The output sequence data were assembled, edited and aligned using Geneious (version 2021.0.3) software, comparing the samples with the reference sequences of *Pvmdr1* (accession number: AY618622), *Pvdhps* (accession number: XM001617159) and *Pvdhfr* (accession number: XM001615032) from GenBank. The known polymorphisms relating to drug resistance at codons 958, 976, 1076 of the *Pvmdr1* gene and codons 57, 58, 61, 117, 173 of the *Pvdhfr* gene, and codons 382, 383, 553, 580 and 558 of *Pvdhps* gene, were evaluated for haplotype. The wild-type haplotypes of *Pvmdr1*, *Pvdhfr* and *Pvdhps* genes were T958/Y976/F1076, F57/S58/T61/R76/S117/I173 and S382/A383/A553/R580/V585, respectively. The mixed alleles were determined according to the emergence of two chromatogram peaks at one nucleotide site through Mutation Surveyor (Soft Genetics LLC., version 5.1, State College, PA, USA). The K10 “AAG” insertion in *Pvcrt-o* gene was determined by comparing with the reference strain of Sal-1 retrieved from *Plasmodium* data base [47]. The nucleotide sequences were submitted to GenBank under accession numbers MZ819186-MZ819695. SAS software (SAS Institute Inc, Version 9.2, Cary, NC, USA) was used for data processing and statistical analysis. The chi-squared test and Fisher’s exact test were used to evaluate differences among the different subgroups. *P* value < 0.05 were used to identify significant differences.

## Results

### Demographics of patients

A total of 149 blood samples from patients with *P. vivax* infections were collected before treatment from hospitals and clinics in Laiza city in 2020. All the patients were confirmed to be infected with *P. vivax* by microscopic examination of Giemsa-stained thick smears through passive case detection. They were treated for 3 days with CQ plus 14 days with PQ according to the anti-malarial drug policy in Myanmar. The majority of the patients were male (70.5%, 105/149) and aged 21–30 years (61.7%, 92/149). The median (range) age of the 149 patients was 22 years (ranges: 6–79) (Additional file 2).

### Prevalence of *Pvmdr1* mutations and K10-insertion in *Pvcrt-o*

**Fig. 2:**
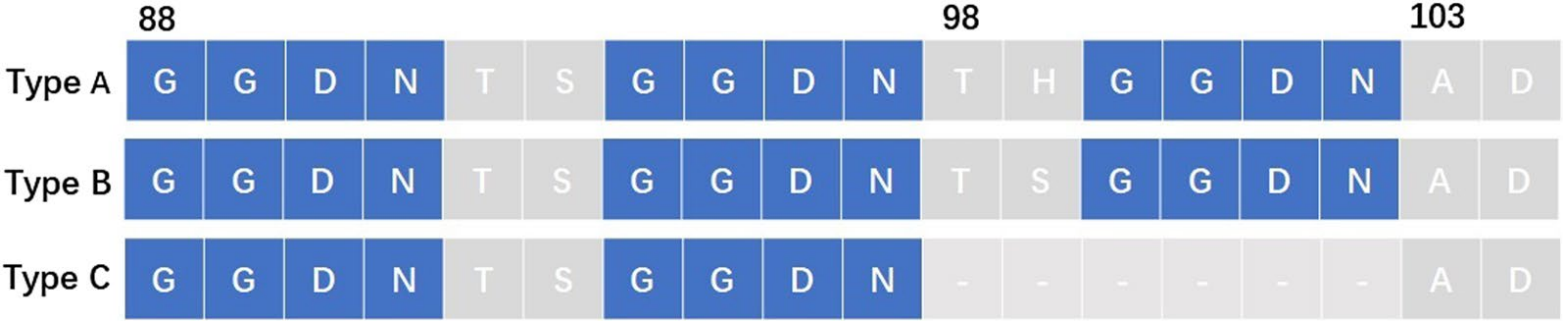
Tandem repeat variation between amino acid positions 88 and 103 in the *Pvdhfr* gene

Among the 149 samples, 82.6% (123/149) samples showed successful amplification of *Pvmdr1*. Mutations in codons 958 and 1076 were identified, with a prevalence of 100% and 52.0% (Fig. 2), respectively. No single nucleotide polymorphism (SNP) was present in codon 976. Analysis of the *Pvmdr1* haplotype prevalence showed that all the isolates were of the mutant type. Appropriately half of them carried a single T958M mutation or a double T958M/F1076L mutation, with a prevalence of 48.0% and 52.0%, respectively (Table 1). The percentage of 88.6% (132/149) samples were successfully sequenced in *Pvcrt-o* gene, and K10 “AAG” insertion was not detected in any of the 132 successfully sequenced samples (Table 1).

**Table 1:** Frequency of alleles with single or multiple mutations in the *Pvmdr1*, *Pvdhfr*, *Pvdhps* and *Pvcrt-o* genes from *P. vivax* isolates

Mutations	Single/multiple mutations	Numbers (%)	*P* value^#^
*Pvmdr1*			< 0.0001
Wild type (T958/Y976/F1076)		0	
Y976F	Single mutation	0	
T958M	Single mutation	59 (48.0)	
T958M + F1076L	Double mutations	64 (52.0)	
Pvdhfr*			< 0.0001
Wild type (F57/S58/T61/S117/I173)		57 (46.3)	
F57I/L^a^	Single mutation	0	
S117T	Single mutation	1 (0.8)	
S58R + S117N	Double mutations	31 (24.4)	
S58R + T61M + S117T	Triple mutations	2 (1.6)	
S58R + T61M + S117N	Triple mutations	1 (0.8)	
F57I + S58R + T61M + S117T	Quadruple mutations	34 (26.8)	
F57L + S58R + T61M + S117N	Quadruple mutations	1 (0.8)	
*Pvdhps*			< 0.0001
Wild type (A383/A553)		103 (78.0)	
A383G	Single mutation	0	
V585A	Single mutation	0	
A553G	Single mutation	2 (1.5)	
A383G + A553G	Double mutations	27 (20.5)	
*Pvcrt-o*			
Wild type (Without K10 insertion)		132 (100.0)	

* Among 32 samples, those with deletion of GGDN repeats from position 95 to 99 were not included. The I13L and I173L mutant alleles were not detected

^a^ A total of 39 samples with mutations in codon 57 including 34 samples carrying 57I and 4 carrying 57L, but F57I/L only existed in quadruple mutations

^#^ The chi-squared test or Fisher’s exact test was used to evaluate differences among the frequency of alleles of each gene

### Polymorphisms in the *Pvdhfr* gene

The *Pvdhfr* amplicon was successfully sequenced for 85.2% (127/149) *P. vivax* isolates. Amino acid changes in *Pvdhfr* due to mutations in codons 57, 58, 61, 99 and 117 were detected in 29.9%, 54.3%, 27.6%, 44.9% and 55.1% of the samples, respectively. In addition, the I13L and I173L was not detected in present study. The wild type was predominant (46.3%, 57/127). The two most common point mutations were S58R (54.3%) and S117T/N (55.1%), followed by H99S (44.9%), F57L/I (29.9%), and T61M (27.6%). Among 70 isolates that carried a point mutation in codon 117, 38 carried 117T whereas 32 carrying 117 N. Moreover, 38 samples with a point mutation in codon 57 included 34 isolates carrying 57I and 4 carrying 57 L. A single mutation in codon 117 was identified in only one isolate. In addition, one synonymous mutation in codon 69, TAT to TAC (Y), was detected in two isolates.

The haplotype analysis revealed seven distinct allelic forms of the *Pvdhfr* gene (Table 1); among them, the wild type was predominant. A quadruple-mutant haplotype, F57I/S58R/T61M/S117T (26.8%) and a double-mutant haplotype S58R/S117N (24.4%) were the two most common mutant types (Fig. 2). The single-mutant haplotype S117T, triple mutant types S58R/T61M/S117N and S58R/T61M/S117T and quadruple-mutant haplotype F57I/S58R/T61M/S117N were rare, being identified in only one, two, one, and one isolate, respectively.

Variations were identified in the central tandem repeat region between amino acid positions 88 and 103 of *Pvdhfr* (Fig. 2). Three types of tandem repeat variation were observed: Type A contained three copies of GGDN repeats, which was the same as the reference strain (accession number: X98123.1) designated as the wild type [48]; Type B also had three copies of GGDN repeats but showed a mutant allele in codon 99; Type C lacked six amino acids from positions 98 to 103.

### Polymorphisms in the *Pvdhps* gene

A percentage of 88.6% (132/149) samples were successfully assessed for the *Pvdhps* gene. *Pvdhps* displayed limited polymorphisms and a relatively low prevalence of mutations. Nonsynonymous mutations were detected only in codons 383 and 553, with a prevalence of 20.5% (27/132) and 22.0% (29/132), respectively. The wild-type *Pvdhps* was dominant (78.0%) (Table 1; Fig. 2). Among the mutant types, the single-mutant A553G was rare and only detected in two isolates, whereas the double-mutant A383G/A553G was more frequent (20.5%, 27/132) (Table 1). Point mutations in codons 382, 580 and 585 were not observed in this study.

### Analysis of allelic combinations of *Pvdhfr* and *Pvdhps*

Allelic combinations of the *Pvdhfr* and *Pvdhps* enzymes are responsible for the biosynthesis of folate and are potentially under similar drug pressure. A total of 118 isolates were sequenced successfully for both genes. The combinations of point mutations in the *Pvdhfr* and *Pvdhps* genes were analyzed in codons 57, 58, 61, and 117, and codons 383 and 553, respectively (Table 2). Eleven haplotypes were identified, and 33.9% (40/118) harboured the wild type, as the predominant allele. The double *Pvdhfr* mutant S58R/S117N and wild-type *Pvdhps* was the most common mutant haplotype, with a frequency of 24.6% (29/118). The quadruple-mutant F57I/S58R/T61M/S117T/WT, quadruple *Pvdhfr* mutant F57I/S58R/T61M/S117T and wild-type *Pvdhps* alleles or double *Pvdhps* mutant A383G/A553G were present at intermediate frequencies of 16.1%, 11.0% and 9.3%, respectively. Rare haplotypes including single-, triple-, quadruple-, and quintuple-mutant alleles, were detected in one isolate each (Table 2).

**Table 2:** Combination of *Pvdhfr* and *Pvdhps* mutations in *P. vivax* isolates from northeastern Myanmar

Haplotypes *Pvdhfr/Pvdhps*	*Pvdhfr*				*Pvdhps*		Number (%)
	F57I/L	S58R	T61M	S117T/N	A383G	A553G	
WT/WT	F	S	T	S	A	A	40 (33.9)
WT/A553G	F	S	T	S	A	**G**	1 (0.8)
WT/A383G + A553G	F	S	T	S	**G**	**G**	11 (9.3)
S58R + S117N/WT	F	**R**	T	**N**	A	A	29 (24.6)
F57L + S58R + S117N/WT	**L**	**R**	T	**N**	A	A	1 (0.8)
F57I + S58R + T61M + S117T/WT	**I**	**R**	**M**	**T**	A	A	19 (16.1)
F57L + S58R + T61M + S117T/WT	**L**	**R**	**M**	**T**	A	A	1 (0.8)
F57L + S58R + S117T/A553G	**L**	**R**	T	**T**	A	**G**	1 (0.8)
S58R + S117N/A383G + A553G	F	**R**	T	**N**	**G**	**G**	1 (0.8)
F57L + S58R + S117T/A383G + A553G	**L**	**R**	T	**T**	**G**	**G**	1 (0.8)
F57I + S58R + T61M + S117T/A383G + A553G	**I**	**R**	**M**	**T**	**G**	**G**	13 (11.0)
Total							118

WT: wild type

## Discussion

Myanmar has the heaviest malaria burden in the GMS [49], and its geographical location between Southeast Asia and South Asia makes it a strategically important point for the potential spread of resistant parasites. In the present study, genetic polymorphisms in the *Pvdhps* and *Pvdhfr* genes associated with SP resistance and *Pvcrt-o* and *Pvmdr1*, two putative molecular markers of resistance to CQ, were analysed to evaluate the level of drug resistance in northeastern Myanmar.

Previous studies have shown that 48.3–72.7% of isolates carried *Pvcrt-o* K10 insertions in Myanmar between 2009 and 2016 [21, 34], and approximately 19% of the samples from Yunnan Province bordering Myanmar harbored this insertion [50]. In the present study, among all the tested samples, the K10 “AAG” insertion in the *Pvcrt-o* gene was not identified, in contrast to the results of previous studies. However, these results are validated to some extent with the high CQ cure rate for *P. vivax* along the China-Myanmar border [37, 51]. Interestingly, the K10 insertion is rarely observed in Thailand, the border region between Thailand and Myanmar, and the border region between Thailand and Cambodia [44, 52]. Other studies have reported no association between the K10 insertion and in vitro or ex vivo *P. vivax* CQ resistance [22, 53, 54]. Given the geographical genetic differences among parasite populations, the prevalence of the *Pvcrt-o* K10 insertion shows significant temporal and spatial heterogeneity [55] and the discrepancy may have resulted from differences in geography, population movement or sample size issues. Moreover, the role of the *Pvcrt-o* K10 insertion in CQ resistance in the parasite remains unknown.

*Pvmdr1* T958M mutation is present in isolates from different countries having low to high levels of CQ resistance, thus T958M appears to be an allelic variant of the wild type and is most likely not associated with CQ resistance [24, 56]. In this study, *Pvmdr1* T958M was present in all the tested samples, whereas F1076L occurred at an intermediate frequency, with a prevalence of 52.0%. However, Y976F was not detected in this study, although it has been frequently reported in other regions with a high prevalence [18, 21–23, 53, 57]. Another study also found that Y976F was rare in Myanmar [34], which supports the findings of this study. In addition, the association of the *Pvmdr1* substitution Y976F with CQ-resistant *P*. *vivax* in vitro was confirmed in Papua, Indonesia [53], while other studies did not find such an association [24].

*Pvdhfr* and *Pvdhps*, the targets of SP drugs, disrupt folate synthesis in *P. vivax* [12, 14, 25]. Compared with *Pfdhfr* and *Pfdhps* in *P. falciparum*, *Pvdhfr* and *Pvdhps* are highly conserved in *P. vivax* [58]. The prevalence of the *Pvdhfr* mutation (53.7%) was lower in this study than in other studies from the China-Myanmar border, southern Thailand and western Myanmar [23, 44, 50, 52]. Moreover, amino acid changes in PvDHFR were detected in 27.6–55.1% of the isolates, which was also lower than the values in other studies [34, 44, 52]. With respect to *Pvdhfr* haplotypes, the double-mutant S58R/S117N and quadruple-mutant F57I/S58R/T61M/S117T were dominant in northeastern Myanmar and were also the most common types in Myanmar, Thailand and southern Yunnan Province, China [50, 52]. A previous study on the expression of mutant PvDHFR-TS in a yeast system demonstrated that these mutations confer high levels of pyrimethamine resistance, and the quadruple mutation resulted in a thousand-fold increase in resistance to pyrimethamine [17]. Interestingly, some researchers have hypothesized that the T61M mutation may be a compensatory mutation to offset possible fitness costs resulting from the carriage of other resistance mutations, because a triple *Pvdhfr* mutant (58R/61M/117T) resulted in susceptibilities similar to those of the wild-type to all drugs with the exception of pyrimethamine, for which this haplotype conferred a 58-fold increase in resistance. In this study, single mutations at residues 58 and 117 were rare, as was the triple mutation, but the double mutation S58R/S117N was more common. These results suggest that vivax parasites in northeastern Myanmar may be under low to medium drug pressure. In addition, the majority (74.8%) had three copies of tandem repeats in *Pvdhfr* gene and Type B was predominant (44.9%, 57/127), followed by Type A (29.9%) and Type C (25.2%). This pattern was different from that reported from southern China [50], which showed Type C as the main type (40.5%, 118/291), followed by Type B and Type A. However, the mechanism of action of the tandem repeat variants in pyrimethamine resistance remains unclear [44], as these variants are not present in the *Pfdhfr* gene in *P. falciparum* [59, 60].

Similar to SP resistance in *P. falciparum*, mutations in *Pvdhps* alone are thought to be insufficient to confer resistance to SP. Strains containing both the *Pvdhps* A383G and A553G mutations and mutant *Pvdhfr* alleles have been implicated in SP treatment failure [61]. The *Pvdhps* mutations exhibited much less diversity and lower prevalence in this study than in previous studies, which reported a high mutation prevalence (80–90%) in *Pvdhps* in Malaysia, Thailand, India, Indonesia, and China and high failure rates of SP treatment [14, 52, 62–64]. Additionally, the prevalence of *Pvdhfr* mutations in regions where *P. vivax* is the dominant parasite is lower than that in regions where *P. falciparum* and *P. vivax* are co-endemic; the reason for this may be that the pressure of SP treatment of *P. falciparum* can lead to co-selection of *Pvdhfr* mutant alleles, which could also occur for other drugs, including artemisinin partner drugs [63].

Recently, high treatment efficacy of CQ was found in Bangladesh, Bhutan, India and Nepal, but high failure rates of treatment with CQ were confirmed in some sites in Myanmar and Timor-Leste [65]. However, this study demonstrated that the frequency of mutant haplotypes in northeastern Myanmar was relatively low, whereas the frequency of the wild type was increasing. In addition, genetic polymorphisms were rarer than in other regions of Myanmar, as shown in previous reports as well. This result is consistent with the treatment efficacy of CQ reported from northeast Myanmar [35] and the China-Myanmar border [51]. These variations in drug resistance genes may suggest different drug selection pressures imposed on local *P. vivax* populations. The factors that influence the evaluation of drug resistance are not completely known, but drug pressure is probably a key element for the selection of resistant parasite mutants.

This study had limitations. First, *Pvcrt-o* and *Pvmdr1* are only putative markers of CQ resistance and the *Pvmdr1* copy number was not evaluated in this study. There was limited evidence for CQ resistance without in vitro susceptibility test of *P. vivax* isolates and in vivo therapeutic efficacy study to highlight therapeutic failure. Second, samples were collected only from Laiza city through passive case detection, which may not describe the whole picture of drug resistance in this region.

## Conclusions

GMS has become a focus in the war against malaria after the emergence of artemisinin-resistant falciparum malaria, especially in remote areas along the Myanmar border. This study identified genetic polymorphisms in genes associated with drug resistance in *P. vivax* parasites in northeastern Myanmar, which indicated the risks associated with drug resistant *P. vivax* phenotypes, especially antifolate resistance. Therefore, continued surveillance of anti-malarial drug resistance markers is needed in northeastern Myanmar to inform case management and update treatment guidelines.

## Supplementary Information

**Additional file 1: Table S1.** Primers and cycling conditions for amplification of *Pvcrt-o*, *Pvmdr1*, *Pvdhps* and *Pvdhfr* by a PCR assay.

**Additional file 2: Table S2.** Demographics of patients included in this study.

## Data Availability

The nucleotide sequences were submitted to GenBank under accession numbers MZ819186-MZ819695. The datasets analysed in this study are available from the corresponding author on reasonable request.
