# Supplementary material for: Genetic polymorphisms in genes associated with drug resistance in Plasmodium vivax parasites from northeastern Myanmar
Source: Malar J. 2022 Mar 3;21:66. doi: 10.1186/s12936-022-04084-y (PMC8892751; doi:10.1186/s12936-022-04084-y)
Supplement: Supplementary file 1 — Additional file 1: Table S1. Primers and cycling conditions for amplification of Pvcrt-o, Pvmdr1, Pvdhps and Pvdhfr by a PCR assay. [file 12936_2022_4084_MOESM1_ESM.docx]

**Table S1. Primers and cycling conditions for amplifying *Pvcrt-o*, *Pvmdr1*, *Pvdhps* and *Pvdhfr* by PCR assay**

| **Genes** | **Primer 5’→3’** | **Cycling conditions** | **Product size (bp)** | **Reference** |
| --- | --- | --- | --- | --- |
| *Pvcrt-o* | F: TCCTTGCCGCTGATTCTACG  R: GGTAACGTTCATCGGGGGTT | 95°C 10min, [95°C 30s, 60°C 45s, 72°C 1min]×35 cycles, 72°C 10min | 327 | Nyunt et al., 2017 |
| *Pvmdr1* (1st) | F: ACGACATGATCCAAACGACA  R: CTTATATACGCCGTCCTGCAC | 94°C 5min, [94°C 30s, 60°C 30s, 68°C 3min]×20 cycles, 68°C 5min | 2729 | Barnadas et al., 2011 |
| *Pvmdr1* (2nd) | F: GGATAGTCATGCCCCAGGATTG  R: CATCAACTTCCCGGCGTAGC | 94°C 5min, [94°C 30s, 60°C 30s, 68°C 3min]×40 cycles, 68°C 5min | 604 | Lu et al., 2011 |
| *Pvdhps* (1st) | F: GATGGCGGTTTATTTGTCG  R: GCTGATCTTTGTCTTGACG | 94°C 5min, [94°C 30s, 58°C 30s, 68°C 1min]×20 cycles, 68°C 5min | 979967 | Ding et al., 2003 |
| *Pvdhps* (2nd) | F:GCTGTGGAGAGGATGTTC  R: CCGCTCATCAGTCTGCAC | 94°C 5min, [94°C 30s, 58°C 30s, 68°C 45s]×40 cycles, 68°C 5min | 731 |  |
| *Pvdhfr* (1st) | F: CACCGCACCAGTTGATTCCT  R: CCTCGGCGTTGTTCTTCT | 94°C 5min, [94°C 30s, 58°C 30s, 68°C 1min]×20 cycles, 68°C 5min | 979 | Ding et al., 2003 |
| *Pvdhfr* (2nd) | F: CCCCACCACATAACGAAG  R: CCCCACCTTGCTGTAAACC | 94°C 5min, [94°C 30s, 58°C 30s, 68°C 45s]×40 cycles, 68°C 5min | 755 |  |
