# Supplementary material for: Genetic polymorphisms in genes associated with drug resistance in Plasmodium vivax parasites from northeastern Myanmar
Source: Malar J. 2022 Mar 3;21:66. doi: 10.1186/s12936-022-04084-y (PMC8892751; doi:10.1186/s12936-022-04084-y)
Supplement: Supplementary file 2 — Additional file 2: Table S2. Demographics of patients included in this study. [file 12936_2022_4084_MOESM2_ESM.docx]

**Table S2. Demographics of patients included in this study**

| **Sex** | **Age (years)** | | | | | | **Total** |
| --- | --- | --- | --- | --- | --- | --- | --- |
|  | <10 | 10～20 | 21～30 | 31～40 | 41～50 | >50 |  |
| Female | 0 | 16 | 23 | 2 | 0 | 0 | 41 |
| Male | 1 | 25 | 69 | 5 | 0 | 5 | 105 |
| unknown |  |  |  |  |  |  | 3 |
